# Supplementary material for: The New SH3b_T Domain Increases the Structural and Functional Variability Among SH3b-Like CBDs from Staphylococcal Phage Endolysins
Source: Probiotics Antimicrob Proteins. 2024 Jul 30;17(6):3930–43. doi: 10.1007/s12602-024-10309-0 (PMC12634807; doi:10.1007/s12602-024-10309-0)
Supplement: Supplementary file 1 — Supplementary file1 A PDF file containing Supplementary Tables S1 , Supplementary Figs. S1–S3 , and an explanatory caption for Online Resource 2 (PDF 619 KB) [file 12602_2024_10309_MOESM1_ESM.pdf]

Online Resource 1 from:

The new *SH3b_T* domain increases the structural and functional variability among SH3b-like CBDs from staphylococcal phage endolysins

Roberto Vázquez*, Diana Gutiérrez*, Dennis Grimon, Lucía Fernández, Pilar García, Ana Rodríguez, Yves Briers#

Author to whom correspondence should be addressed (yves.briers@ugent.be)

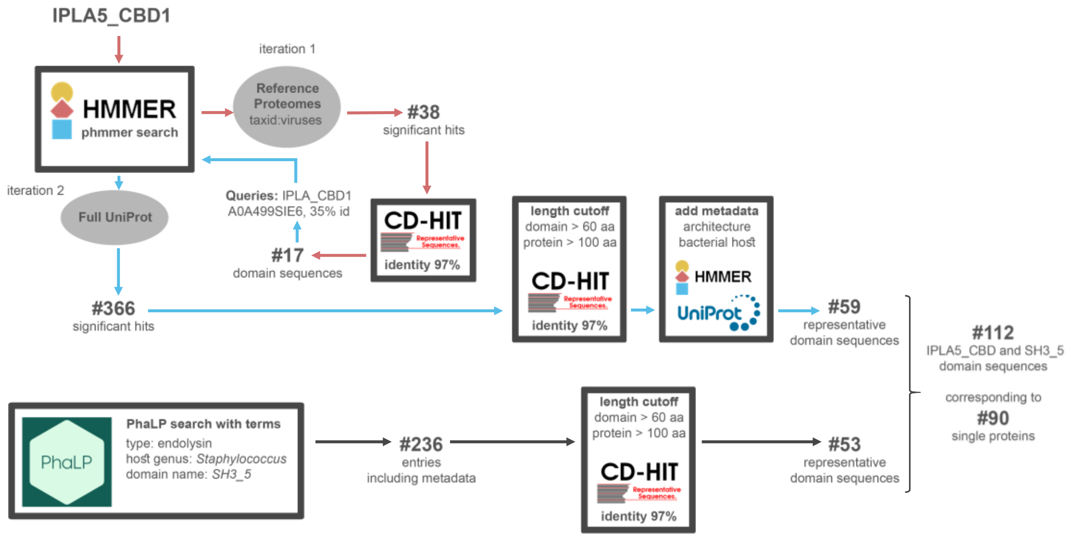

Supplementary Fig. S1. Workflow for constructing the datasets of SH3b-like sequences.

a

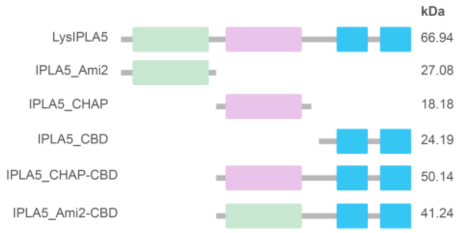

b

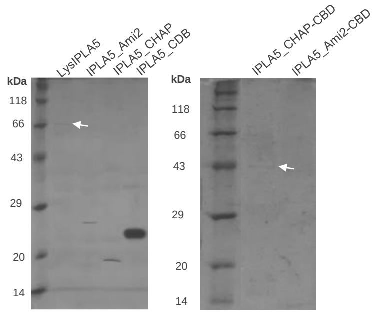

c

	Coagulase negative streptococci													Coagulase positive streptococci					
	S. epidermidis								*S. hominis*	*S. xylosus*	*S. haemolyticus*	*S. gallinarum*	*S. kloosii*	*S. aureus*					
	**F12**	**B**	**DG2n**	**YLIC13**	**LO5RB1**	**ASLD1**	**DH3LIK**	**Z2LD14**	**ZL31-13**	**ZL61-2**	**ZL89-3**	**ZL90-5**	**ZL74-2**	**Sa9**	**IPLA1**	**IPLA16**	**15981**	**V329**	**MRSAE10**
**IPLA5_CHAP-CBD**	0.75	0.75	0.75	0.75	0.75	0.75	0.75	0.75	0.75	>1.5	>1.5	0.75	0.75	>1.5	>1.5	>1.5	>1.5	>1.5	>1.5
**LysIPLA5**	>1.86																		
**IPLA5_Ami2**	>2.67																		
**IPLA5_CHAP**	>6.09																		
**IPLA5_CBD**	>34.16																		
**IPLA5_Ami2-CBD**	>1.25																		

Supplementary Fig. S2. Functional dissection of LysIPLA5 domains. (a) Scheme of the proteins tested in this experiment. (b) SDS-PAGE depicting the proteins after purification. (c) MIC values calculated for each protein.

Supplementary Table S1. Absolute MIC values (µM) for the proteins and strains shown in Fig. 4.

**Strain**	**RODI_CHAP**	**RODI_CHAP-CBD**	**RODI_CHAP-IPLA5_CBD**	**C1C_CHAP**	**C1C_CHAP-CBD**	**C1C_CHAP-IPLA5_CBD**
S. epidermidis F12	6	1.5	0.1875	3	0.375	0.1875
S. epidermidis B	6	6	0.1875	3	0.375	0.1875
S. epidermidis DG2n	6	6	0.1875	3	0.375	0.09375
S. epidermidis YLIC13	12	6	0.1875	3	0.375	0.1875
S. epidermidis LO5RB1	3	3	0.09375	3	0.0468	0.023438
S. epidermidis ASLD1	3	6	0.1875	6	0.375	0.1875
S. epidermidis DH3LIK	6	3	0.1875	3	0.375	0.1875
S. epidermidis Z2LD14	6	3	0.1875	3	0.375	0.09375
S. hominis ZL31-13	1.5	3	0.1875	1.5	0.09375	0.09375
S. xylosus ZL61-2	6	6	3	3	1.5	1.5
S. haemolyticus ZL89-3	3	6	3	3	1.5	1.5
S. gallinarum ZL90-5	3	1.5	1.5	6	1.5	0.75
S. kloosi ZL74-2	3	1.5	1.5	3	1.5	0.75
S. aureus Sa9	3	0.375	3	3	1.5	1.5
S. aureus IPLA1	1.5	1.5	1.5	6	1.5	1.5
S. aureus IPLA16	3	1.5	1.5	6	1.5	1.5
S. aureus 15981	3	1.5	1.5	6	1.5	1.5
S. aureus V329	3	1.5	1.5	3	1.5	1.5
S. aureus MRSAE10	3	3	1.5	3	3	1.5

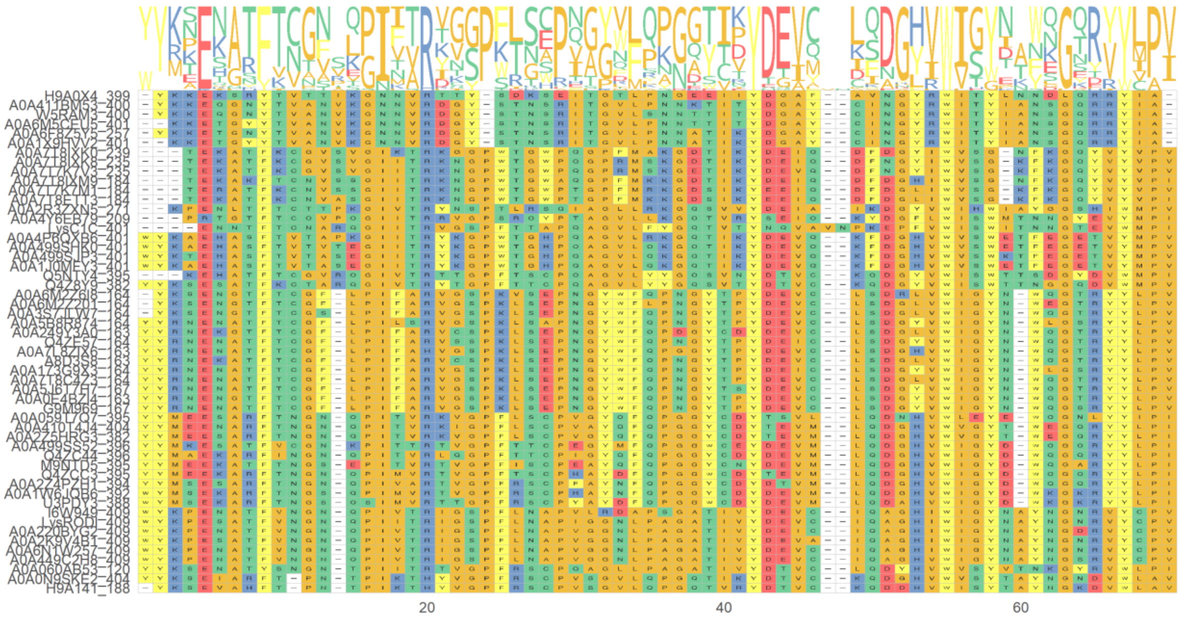

Supplementary Fig. S3. MSA of the *SH3_5* subset of CBD sequences. Sequences are identified in the left side by their unique identifier as available in Online Resource 2. Amino acids are code-colored depending on their chemical nature (non-polar, aliphatic = orange; non-polar, aromatic = yellow; polar, non-charged = green; polar, positively charged = blue; polar, negatively charged = red). On top of the MSA, a logo shows the relative frequency of each amino acid at each position.

Online Resource 2. Curated set of representative sequences from *SH3_5*, *SH3b_T*, *PSA_CBD* and *PBC5_CBD* including sequence identifiers, domain delimitation coordinates and the sequence of the original full endolysins (Available as a separate .csv file).
